# Electrochemical Performance of Nitrogen-Doped TiO_2_ Nanotubes as Electrode Material for Supercapacitor and Li-Ion Battery

**DOI:** 10.3390/molecules24162952

**Published:** 2019-08-14

**Authors:** Tamilselvan Appadurai, Chandrasekar M Subramaniyam, Rajesh Kuppusamy, Smagul Karazhanov, Balakumar Subramanian

**Affiliations:** 1 National Centre for Nanoscience and Nanotechnology, University of Madras, Guindy Campus, Chennai, Tamil Nadu 600 025, India; 2 Department of Fibre and Polymer Technology, KTH Royal Institute of Technology, 11428 Stockholm, Sweden; 3 Department of Physical Chemistry, University of Madras, Guindy Campus, Chennai, Tamil Nadu 600 025, India; 4 Department for Solar Energy, Institute for Energy Technology (IFE), 2027 Kjeller, Norway

**Keywords:** electrochemical anodization, TiO_2_ nanotubes, Nitrogen doping, supercapacitor, Lithium-ion battery

## Abstract

Electrochemical anodized titanium dioxide (TiO_2_) nanotubes are of immense significance as electrochemical energy storage devices owing to their fast electron transfer by reducing the diffusion path and paving way to fabricating binder-free and carbon-free electrodes. Besides these advantages, when nitrogen is doped into its lattice, doubles its electrochemical activity due to enhanced charge transfer induced by oxygen vacancy. Herein, we synthesized nitrogen-doped TiO_2_ (N-TiO_2_) and studied its electrochemical performances in supercapacitor and as anode for a lithium-ion battery (LIB). Nitrogen doping into TiO_2_ was confirmed by Raman spectroscopy and X-ray photoelectron spectroscopy (XPS) techniques. The electrochemical performance of N-TiO_2_ nanotubes was outstanding with a specific capacitance of 835 µF cm^−2^ at 100 mV s^−1^ scan rate as a supercapacitor electrode, and it delivered an areal discharge capacity of 975 µA h cm^−2^ as an anode material for LIB which is far superior to bare TiO_2_ nanotubes (505 µF cm^−2^ and 86 µA h cm^−2^, respectively). This tailor-made nitrogen-doped nanostructured electrode offers great promise as next-generation energy storage electrode material.

## 1. Introduction

Lithium-ion batteries (LIBs) and supercapacitors are the best known electrochemical energy storage (EES) devices for their high energy density (kW h kg^−1^) and power density (kW h^−1^), respectively. LIBs have found applications in our day-to-day electronic devices while supercapacitor-based trams and buses are being tested in a few countries [1]. The existing research progress on the materials chemistry of LIBs and supercapacitors are in focus to replace the fossil fuel-based internal combustion (IC) engine with plug-in/hybrid electric vehicles. Another important prospect is to store and provide electricity when it is needed inorder to minimize the transmission loss and maximize power utilizations. LIBs possess low self-discharge, high gravimetric and volumetric density (W h L^−1^), while a supercapacitor exhibits high charge-discharge rate, power density, and long cyclic performance of over 10,000 cycles [1,2,3,4,5].

TiO_2_ is considered as an alternative anode material that could potentially substitute commercialized graphite. Some of its merits are: higher Li insertion potential (~1.7 V vs. Li^+^/Li^0^), prohibiting lithium plating/dendrite growth (proven safety concern), fast lithium insertion/extraction, low volume change, being environmentally friendly, chemically stable, and having a low cost [6,7,8]. Additionally, it offers theoretical capacity of 332 mA h g^−1^ [9], but due to its poor electronic conductivity (~1 × 10^−12^ S cm^−1^ to 1 × 10^−7^ S cm^−1^) and low Li-ion diffusion (~1 × 10^−15^ cm^2^ s^−1^ to 1 × 10^−9^ cm^2^ s^−1^), the ability of TiO_2_ has affected the lithium-ion storage capacity, which possibly limits its practical use. Therefore, there is a need to develop nanostructures of TiO_2_ with short diffusion length for electronic and Li^+^ transport, increasing the contact area between electrode and electrolyte, and better accommodation of the strain during Li insertion/extraction [10,11]. TiO_2_ nanotubes achieved the above properties, especially, when directly grown from Ti metal foil by the electrochemical anodization technique and, furthermore, displayed an additional advantage such as additive free electrode [12]. However, recent research is driven to tailor the properties of TiO_2_ nanotubes to improve the electronic conductivity and ionic diffusion by making composites with carbon materials [13], metal oxides [14], and doping with nitrogen [15] to increase its electrochemical activity.

Depending upon experimental conditions, nitrogen doping in TiO_2_ (N-TiO_2_) phases leads to different forms such as nitrogen substitution to oxygen atoms, or interstitial NO^2-^, or surface adsorbed N_2,_ etc [16]. Some studies found that ammonia-treated TiO_2_ exhibits the occurrence of interstitial atoms and it form N-Ti-O [17]. Therefore, nitrogen substitution in TiO_2_ causes a reduction and as a result Ti^3+^ and oxygen vacancy are formed. This formation creates additional electrons in the structure and leads to the increase of electronic conductivity [18]. Recently, more studies were carried out on nitrogen-doped 1-D TiO_2_ nanostructures in wide range of potential applications especially in energy storages devices. Hyungku Han et al. [19], reported the performance of LIB with nitrated TiO_2_ hollow nanofibers, which had been prepared by the electrospinning method and demonstrated an excellent improvement in the rate capability and exhibited discharge capacity of 85 mA h g^−1^, which was nearly twice as that of 45 mA h g^−1^ (bare TiO_2_). Hydrothermally prepared N-TiO_2_ nanotubes/graphene composites presented the discharge capacity of 369 mA h g^−1^ at 0.1 Ag^−1^ as better performance in LIBs [20]. N-TiO_2_-B nanowires have been studied for LIB as anode materials and they exhibit enhanced electrochemical performance of 153 mA h g^−1^ at 20 C with a 76% capacity retention even after 1000 cycles, which make them potential candidate in a high-power lithium battery [21]. However, it is valuable that TiO_2_ nanotubes prepared by electrochemical anodization technique have in particular shown additional advantages over other synthesis techniques, particularly, in energy storage devices. These include: (i) titanium itself acts as a current collector and, therefore, minimizes the resistance between the active materials and the current collector; and (ii) enables binder free and conducting agents free active materials. Due to these advantages, anodized TiO_2_ nanotubes can be effectively used as a Li-ion battery anode material.

As the nitrogen-doped anodized TiO_2_ exhibits superior electrochemical performance, this work attempts to report on the comparative performances of rate capability, specific capacitance, cyclic stability, and specific capacity of TiO_2_ nanotubes and N-TiO_2_ nanotubes for both LIB and supercapacitor applications.

## 2. Results and Discussions

### 2.1. Structural Analysis

#### 2.1.1. X-ray Diffraction (XRD)

**Figure 1:**
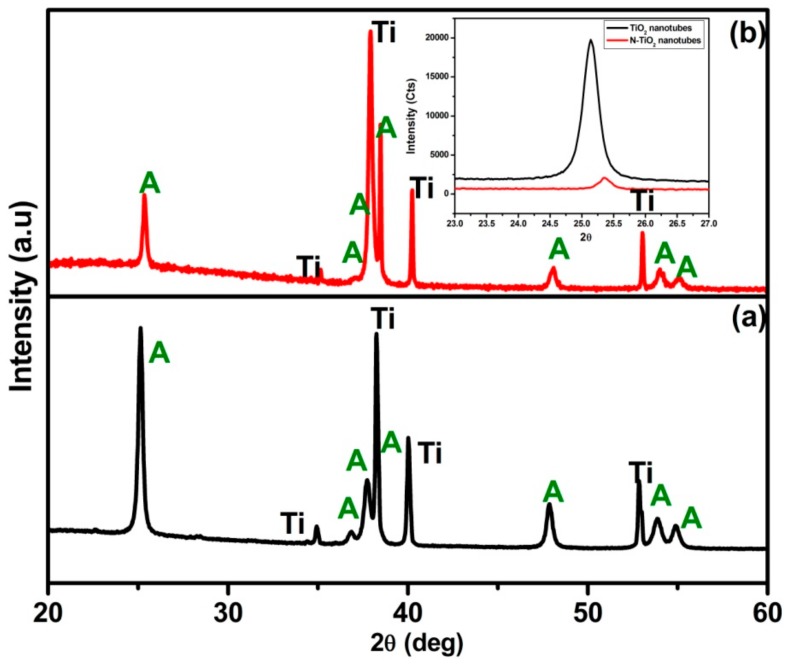
X-ray diffraction (XRD) pattern of (**a**) TiO_2_ nanotubes and (**b**) N-TiO_2_ nanotubes. Inset contains magnified view between 23–27° (2θ Position).

The XRD patterns of TiO_2_ nanotubes annealed at 450 °C for 3 h in (a) air and (b) NH_3_ atmosphere, respectively, are presented in Figure 1. The diffraction peaks corresponding to highly crystalline TiO_2_ anatase phase along with Ti metal peaks were observed for TiO_2_ nanotubes (JCPDS data file no: 89-4921) with no sign of rutile phase, which is in good agreement with reported literature [22]. However, TiO_2_ nanotubes treated in NH_3_ atmosphere, that is, the N-TiO_2_ nanotubes displayed a dominant anatase phase with some significant difference in diffraction patterns compared to TiO_2_ nanotubes that included: (i) decreased intensity; (ii) peak broadening, and (iii) peaks shifting, which is clearly shown inset in Figure 1. In the N-TiO_2_ nanotubes, above mentioned change in XRD could be attributed to the TiO_2_ nanotubes treated in NH_3_ atmosphere resulting in nitrogen substitution into TiO_2_ that induces structural changes in the lattice that prompted peak shifting and broadening and decreased intensity when compared to the TiO_2_ nanotubes [23].

#### 2.1.2. Raman Spectroscopy

**Figure 2:**
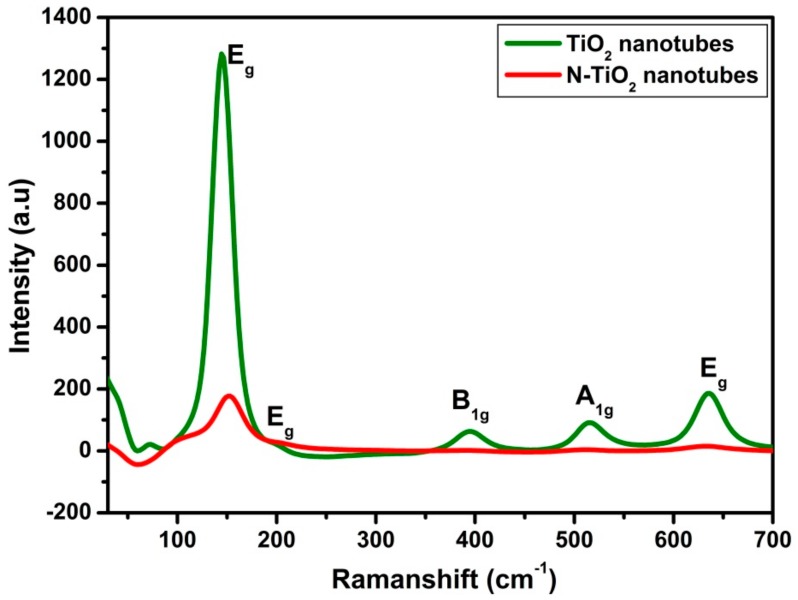
Raman spectra of TiO_2_ nanotubes and N-TiO_2_ nanotubes.

Furthermore, in order to distinguish, the TiO_2_ nanotubes and N-TiO_2_ nanotubes, structural analysis was carried out using Raman spectroscopy as shown in Figure 2. According to the reported data [24], TiO_2_ anatase phase would predominantly display a characteristic line of six fundamental modes that includes A_1g_ (519 cm^−1^), B_1g_ (399 cm^−1^ and 519 cm^−1^) and E_g_ (144 cm^−1^, 197 cm^−1^, and 639 cm^−1^). Herein, TiO_2_ nanotubes spectra showed the presence of active modes peaks at 144.3 cm^−1^, 395.8 cm^−1^, 515.7 cm^−1^ and 636 cm^−1^, which directly confirms the pure anatase phase and no other peaks of rutile phase. In N-TiO_2_ nanotubes, the strongest E_g_ mode at 150 cm^−1^ was clearly visible and could be ascribed to the external vibration of the anatase phase. When compared to TiO_2_ nanotubes, the N-TiO_2_ nanotubes spectra exhibit weak intensity, and shifting toward high frequency, which clearly confirms the nitrogen substitution into the TiO_2_ lattice, which is in good agreement with the reported literatures [25].

### 2.2. Morphological and Compositional Analysis: Field Emission Scanning Electron Microscopy (FESEM) and Energy Dispersive Spectroscopy (EDS)

**Figure 3:**
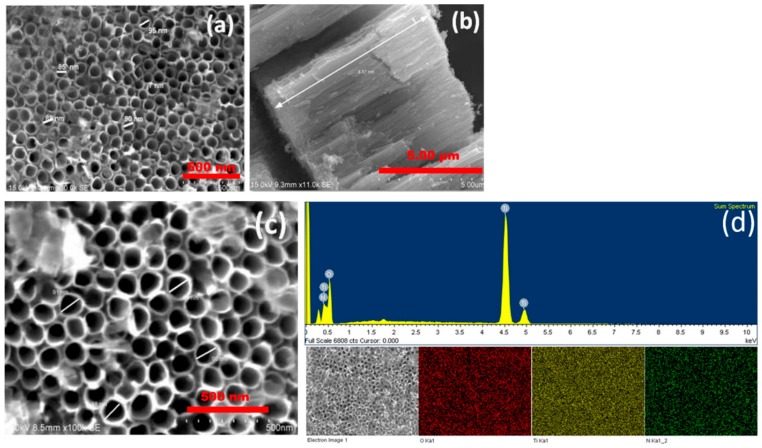
Field emission scanning electron microscopy (FESEM) micrograph of (**a**) top view-TiO_2_ nanotubes, (**b**) cross sectional view-TiO_2_ nanotubes, (**c**) top–view-N-TiO_2_ nanotubes and (**d**) energy dispersive spectroscopy (EDS) of N-TiO_2_ nanotubes.

Topographical views and cross-sectional views of TiO_2_ nanotubes and N-TiO_2_ nanotubes were carried out by FESEM as shown in Figure 3. It is clearly observed that both TiO_2_ nanotubes treated in air and NH_3_ atmosphere have displayed homogenous nanotube morphology. Since, both TiO_2_ nanotubes were synthesized at the same anodization conditions, but annealed in different atmospheres, they do not show any noticeable change in pore diameter. Figure 3d shows the presence of nitrogen in the N-TiO_2_ nanotubes as confirmed by EDS elemental analysis.

### 2.3. Chemical Analysis: X-ray Photoelectron Spectroscopy (XPS)

**Figure 4:**
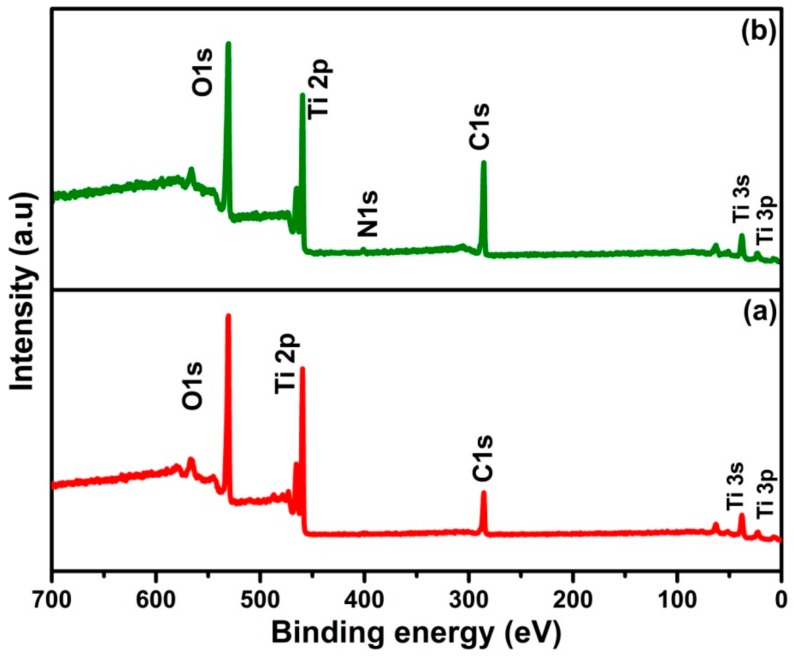
X-ray photoelectron spectroscopy (XPS) survey scan spectra of (**a**) TiO_2_ nanotubes and (**b**) N-TiO_2_ nanotubes.

To investigate the chemical changes that occur during different annealing atmosphere of TiO_2_ nanotubes, XPS measurements were carried out for TiO_2_ nanotubes and N-TiO_2_ nanotubes and the survey scan spectra are shown in Figure 4. The spectra show the presence of elements of Ti, O, and N with trace amounts of carbon in respective samples.

**Figure 5:**
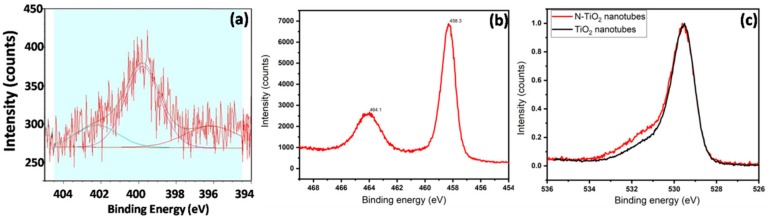
Narrow scan XPS spectra of (**a**) N 1s, (**b**) Ti 2p and (**c**) O 1s of N-TiO_2_ nanotubes.

To investigate the nitrogen doping effect in TiO_2_ nanotubes further, narrow scans of N 1s, Ti 2p, O 1s spectra of N-TiO_2_ nanotubes were measured as shown in Figure 5. The observed core-level N 1s spectra have shown broad range spectrum from 394 eV to 404 eV and it can be interpreted that the N 1s spectra for nitrogen substitutions or interstitial doping, as it still should be a complex process and subject to debate as stated by Asahi et al. [26], and these may be caused by different synthesis procedures adopted by different groups. For spectra, fitting was applied and three peaks of binding energy nearly were exhibited at 396.1 eV, 402.2 eV, and 399.8 eV, which are well in agreement with reported literature [27]. From the narrow scan spectra of Ti 2p of N-TiO_2_ nanotubes (Figure 5b), the peaks at 464.1 eV and 458.3 eV correspond to the 2p_1/2_ and 2p_3/2_, respectively, which clearly indicates the incorporation of nitrogen in TiO_2_ nanotubes [27]. It has been assessed that peaks corresponding to 396.1 eV binding energy were attributed to the substitution of nitrogen to replace lattice oxygen atoms and formation of oxy-nitrides (O–Ti–N) [28] and peaks at binding energy 399.8 eV and 402.2 eV were corresponding to the interstitial doping of nitrogen atoms, and to form bond with oxygen atoms (Ti–O–N), which is well agreement with report literature [29]. This is further confirmed from O 1s narrow scan spectra of TiO_2_ and N-TiO_2_ nanotubes as shown in Figure 5c. While comparing two spectra of O 1s, there are some additional peaks grown in N-TiO_2_ nanotubes at 531.7 eV, and this is due to the interstitial doping of nitrogen into the TiO_2_ lattice. Therefore, the above results confirm the doping of nitrogen atoms into surface of TiO_2_ nanotubes.

### 2.4. Supercapacitor Application

#### 2.4.1. Cyclic Voltammetry (CV)

**Figure 6:**
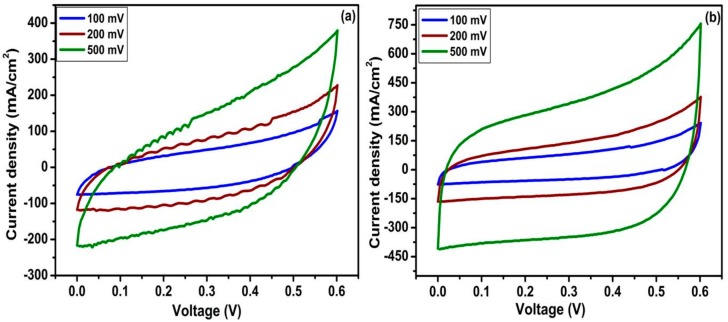
Cyclic voltammetric (CV) curves of (**a**) TiO_2_ nanotubes and (**b**) N-TiO_2_ nanotubes.

The electrochemical performances of TiO_2_ nanotubes and N-TiO_2_ nanotubes as electrode materials for supercapacitor application were carried out by an identical two-electrode system using swagelok cells in an aqueous solution of 1 M KOH. Figure 6 shows the CV curves of TiO_2_ nanotubes and N-TiO_2_ nanotube samples at a scan rate of 100, 200, and 500 mV s^−1^ in the potential window of 0 to 0.6 V. Both CV curves present the typical rectangular shape, which resembles the electrochemical double-layer capacitor (EDLC) as reported in our previous work [30]. Clearly, current density continues to increase as the scan rate increases without any change in curve shape indicating the good rate capability of both samples. From these CV curves, the specific capacitance of both electro-active materials were calculated and found to be 505 µF cm^−2^ for TiO_2_ nanotubes and 835 µF cm^−2^ for N-TiO_2_ nanotubes at a scanning rate of 100 mV s^−1^. Table 1 displays the current density, and specific capacitance as a function of different scanning rates for the TiO_2_ nanotubes- and N-TiO_2_ nanotubes.

**Figure 7:**
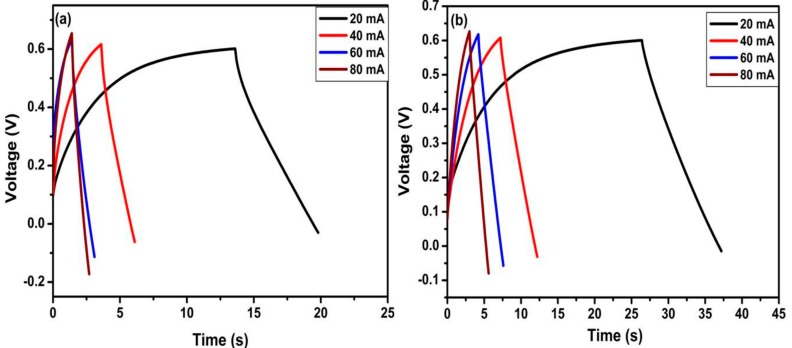
Galvanostatic first charge/discharge curves of TiO_2_ nanotubes and N-TiO_2_ nanotubes.

To further study the electrochemical performance of TiO_2_ nanotubes and N-TiO_2_ nanotubes for supercapacitor applications, galvanostatic charge/discharge measurement were taken at a different current density. Figure 7 shows the first charge/discharge curves of TiO_2_ nanotubes and N-TiO_2_ nanotubes samples at a current density of 80, 160, 240 and 320 µA cm^−2^, which are linear and symmetrical indicating good electrochemical capability and ensuring the electrochemical double layer capacitor behavior [31]. The specific capacitance of the electrode was estimated from the galvanostatic discharge curves according to the following equation:

(1) $$Cs=I\times \left(\frac{\Delta t}{\Delta V}\right)\times A$$

where I represents charge/discharge current (A), Δt is the charge/discharge time (s), ΔV represents the potential window (V), and A represents the electrode area (cm^2^). The specific capacitances of two samples recorded at different current densities have been summarized in Table 2**.** The results obtained reveal the difference between TiO_2_ nanotubes and N-TiO_2_ nanotubes samples. At current density of 160 µA cm^−2^, the TiO_2_ nanotubes sample delivered a specific capacitance of 1508 µF cm^−2^ while N-TiO_2_ nanotubes exhibited overwhelming 3121 µF cm^−2^, which is double the specific capacitance of the former. Therefore, from the aforementioned electrochemical studies, the specific capacitance doubled from N-TiO_2_ nanotubes and could be attributed to its improved electronic conductivity, which facilitates the transport of charge carriers.

#### 2.4.2. Electrochemical Impedance Spectroscopy

**Figure 8:**
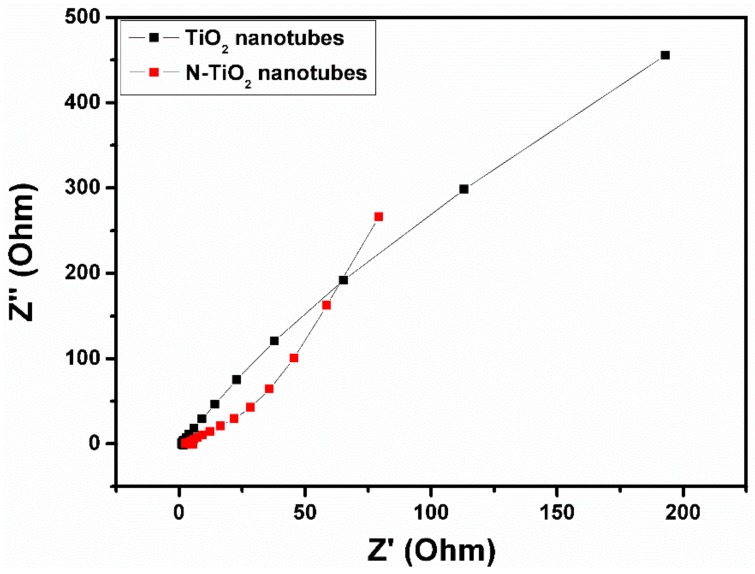
Electrochemical impedance spectra of TiO_2_ nanotubes and N-TiO_2_ nanotubes.

Electrochemical impedance spectroscopy measurements were performed for the TiO_2_ nanotubes and N-TiO_2_ nanotubes at a frequency range from 1 Hz to 1 MHz and its Nyquist plot is shown in Figure 8. From the spectra, it can be seen that both samples could not show any semicircle in the high-frequency region, which indicates good capacitive electrodes. Compared to TiO_2_ nanotubes, low frequency region of N-TiO_2_ nanotubes exhibits clear vertical line, which is due to the ion’s diffusion in the electrolyte to the electrode interface that results in the better performance of the supercapacitor electrode.

Therefore, enhanced capacitive performance of N-TiO_2_ nanotubes could be ascribed to the improved conductivity of the electrode [32].

### 2.5. Lithium-Ion Battery Application

**Figure 9:**
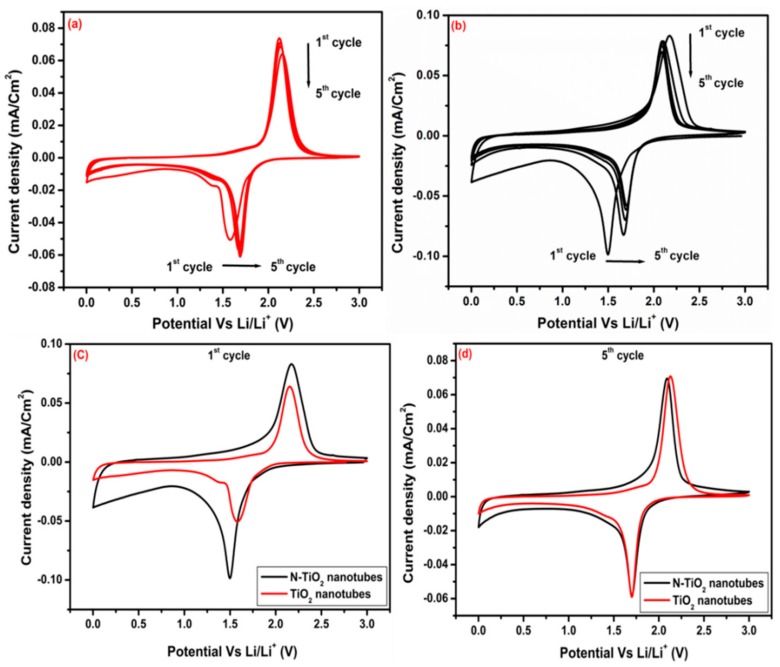
Cyclic voltammetry of (**a**) TiO_2_ nanotubes and (**b**) N-TiO_2_ nanotubes for 5 cycles, respectively, comparing CV of both samples (**c**) 1st cycle and (d) 5th cycle.

To investigate the electrochemical performance of TiO_2_ nanotubes and N-TiO_2_ nanotubes as anode materials for LIB, we have initially recorded cyclic voltammetry for 5 cycles as shown in Figure 9a–d, which were tested under the same conditions and found a significant difference in lithium insertion/extraction during the discharge/charge process between the two samples. CV curves of both samples that were tested at 0.1 mV s^−1^ scan rate, displayed cathodic and anodic peaks, which are associated with Li^+^ intercalation and de-intercalation into TiO_2_ nanotubes. The overall cell reaction for the Li insertion/extraction into TiO_2_ nanotubes can be written as follows:

(2) $$TiO_{2}+x\;{\mathrm{Li}}^{+}+xe^{-}\to Li_{x}TiO_{2}$$

For the first cycle of TiO_2_- and N-TiO_2_ nanotubes samples, cathodic /anodic peaks are located at 1.58/2.15 V and 1.49/2.17 versus Li^0^/Li^+^, respectively, which were attributed to the lithium insertion/extraction from the anatase phase of TiO_2_ and agree well with the peak’s position from reported literature [33]. Here, lithium insertion into TiO_2_ anatase phase is a two-phase process of Li poor (Li_0.01_TiO_2_) and Li rich (Li_0.6_TiO_2_). Table 3 shows the peak position of cathodic, anodic, potential difference of TiO_2_ nanotubes and N-TiO_2_ nanotubes samples. In detailed analysis of CV curves, some clear information has been notified regarding lithiation and delithiation of Li^+^ ions into the TiO_2_ lattice. During 1^st^ cycle, cathodic peaks potential is lower in N-TiO_2_ nanotubes sample compared to TiO_2_ nanotubes, with increasing cycles, the peak position in N-TiO_2_ nanotubes shifted slightly towards higher potential probably due to the activation process for the Li^+^ lithiation in the first cycle, which is in agreement with some of the reported literature [34]. The potential separation between anodic and cathodic peak for N-TiO_2_ nanotubes (0.39 V) is smaller than TiO_2_ nanotubes samples (0.437 V) in the 5th cycle. This reduction in potential difference suggested that N-TiO_2_ nanotubes display high reversibility and faster Li^+^ diffusion [7]. Figure 9c,d, compares the CV curves of the 1st and 5th cycle of TiO_2_ nanotubes and N-TiO_2_ nanotubes samples. It is clear that in the 1st cycle, sweeping area and current density of N-TiO_2_ nanotubes is more than that of TiO_2_ nanotubes, which indicates higher lithium ions storage capabilities and high electrochemical activity. But in the 5th cycle, almost both samples exhibit similar type of behavior and show good reversible capability.

**Figure 10:**
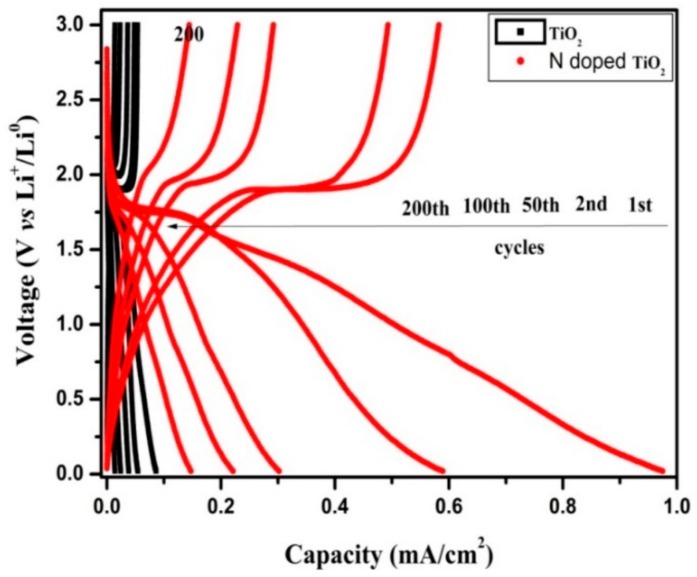
Galvanostatic charge/discharges curves of TiO_2_ nanotubes and N-TiO_2_ nanotubes.

Figure 10 shows the charge/discharge curves in the 1st, 2nd, 50th, 100th, and 200th cycles for the TiO_2_ nanotubes’ and N-TiO_2_ nanotubes’ electrodes between 0 V to 3 V at a current density of 20 µA cm^−2^. Both samples exhibit voltage plateaus that occur at 1.75 V and 1.9 V, which were due to the insertion and extraction of Li^+^ from TiO_2_ structures. The initial discharge capacities of TiO_2_ nanotubes and N-TiO_2_ nanotubes for the 1st cycles were 86 µA h cm^−2^ and 975 µA h cm^−2^, respectively, which indicated that N-TiO_2_ nanotubes exhibit superior performance than the TiO_2_ nanotubes. These can be attributed to accommodation of more Li^+^ in N-TiO_2_ nanotubes due to its increased electronic and ionic conductivity.

**Figure 11:**
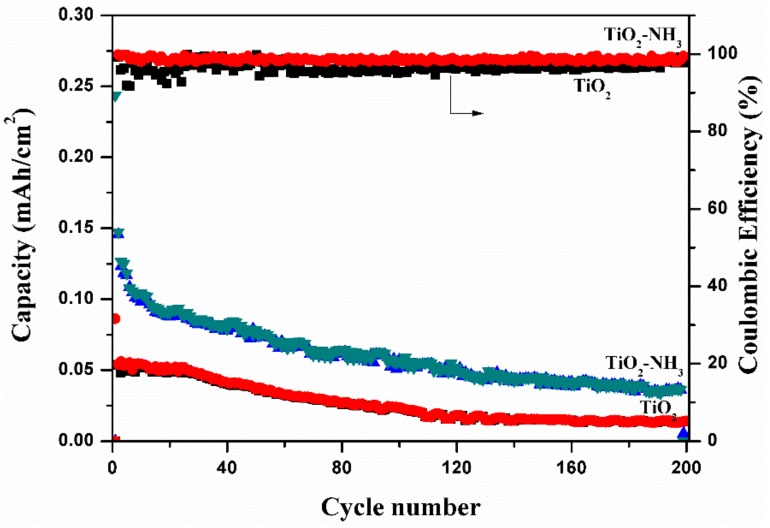
Cyclic performances of TiO_2_ nanotubes and N-TiO_2_ nanotubes.

Figure 11 shows the specific capacities as a function of cycle number plot to understand the long cyclic stability of TiO_2_ nanotubes and N-TiO_2_ nanotubes samples. It is seen that initial reversible discharge capacity of N-TiO_2_ nanotubes and TiO_2_ nanotubes samples are 975 µA h cm^−2^ and 86 µA h cm^−2^, respectively, and with further cycling of 200 cycles, the specific capacity comes down to 145 µA h cm^−2^ and 13 µA h cm^−2^ for N doped TiO_2_ nanotubes and TiO_2_ nanotubes samples, respectively, which is a higher areal capacity compared to other reported TiO_2_ nanotubes based anode materials. For both samples, coulombic efficiency of more than 98% has been achieved even after 200 cycles and as a result it indicates that the nitrogen-doped TiO_2_ nanotubes exhibit superior cyclic performance that can be attributed to the fast Li^+^ diffusion and increased electronic conductivity.

**Figure 12:**
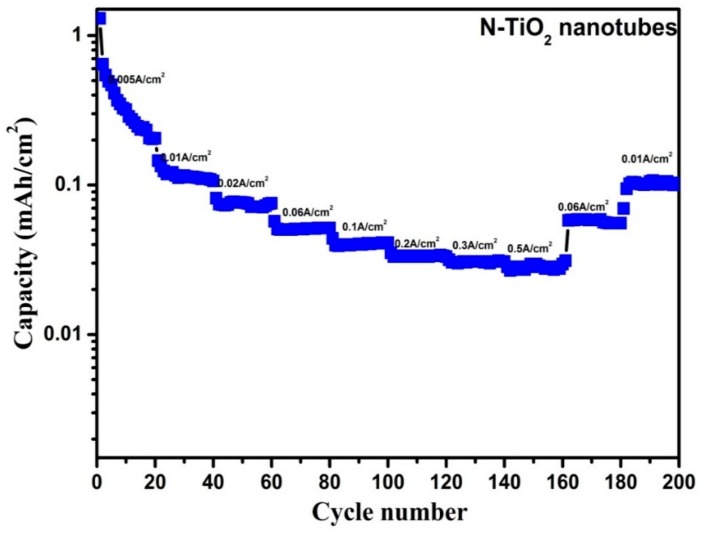
Rate capability performances of N-TiO_2_ nanotubes.

As, N-TiO_2_ nanotubes outperformed the TiO_2_ nanotubes with the highest ever reported areal capacity, we intended to test its rate capability at various current densities from 5 to 500 mA cm^−2^ so as to check its feasibility for high-power applications, which is shown in Figure 12. For these measurements, a fresh cell was made and, therefore, in an initial current density of 5 mA cm^−2^ the capacity (1.3 mA h cm^−2^) fell rapidly due to untreated electrochemical process [35]. For N-TiO_2_ nanotubes, it exhibited reversible capacity of 145.6, 81.6, 57.2, 44.0, 35.2, 31.6 and 28.4 µA h cm^−2^ at a current density of 10, 20, 50, 100, 200, 300, and 500 mA cm^−2^, respectively. For each current density, the capacities were recorded for 20 cycles and it displayed constant capacity except in lower current density. This reveals that discharge capacity of the electrode decreases along with the increase of current densities and this may be attributed to the insulating character of the N-TiO_2_ nanotubes sample. When it reverted back to current density of 50 and 10 mA cm^−2^, it displayed a reversible capacity of 58 and 101.2 µA h cm^−2^, respectively, even after 200 cycles. As a result, the N-TiO_2_ nanotubes sample exhibits superior cyclic performances, which makes it a suitable negative material for LIB.

## 3. Materials and Methods

All chemicals of analytical grade were used without any further purification. This section includes the synthesis of TiO_2_ nanotubes and nitrogen doped by cracking ammonia gas. The as-obtained TiO_2_ nanotubes were physically and electrochemically characterized for LIBs and supercapacitor applications.

### 3.1. Synthesis of TiO_2_ Nanotubes and N-Doped TiO_2_ (N-TiO_2_) Nanotubes

In a typical experimental procedure, TiO_2_ nanotubes were synthesized as reported in our previous work [30], here, the electrolyte consists of 70% ethylene glycol, 30% glycerol, 2% distilled water (DD) water containing 0.3 M ammonium fluoride. For anodization, Ti foils were polished as described in our previous work [30] and to get mirror polish of Ti metal, diamond paste of 3 µm and 0.5 µm were used. Anodization process was carried out at an anodizing voltage of 50 V for 4 h using titanium foil 2 cm × 1 cm as working electrode and platinum foil as counter/reference electrode. As-obtained anodized TiO_2_ nanotubes was washed and air dried at room temperature. To obtain TiO_2_ nanotubes and N-TiO_2_ nanotubes, the samples were further subjected to thermal annealing at 450 °C for 3 h in air and ammonia atmosphere, respectively.

### 3.2. Material Characterizations

All samples were characterized for phase purity and morphology using various sophisticated analytical techniques. Phase purity was analyzed using the X-ray diffraction (XRD) technique with X’PERT PRO PANalytical equipment operated at 1°/min scan rate and 0.02° step size while Raman spectroscopy (Nanophoton Raman-11, Japan) measured at a wavelength of 532 nm line of Nd-YAG laser. Morphology of particle distribution and its elemental compositional were visualized using field emission scanning electron microscopy (FESEM Hitachi, Japan, Model No.: SU6600) operated at 5 kV and 10 µA coupled with energy dispersive spectroscopy (EDS). The chemical composition and N 1s, O1 s and Ti 2p spectra were determined by using an X-ray photoelectron spectroscopy (XPS) instrument (Omicron nanotechnology) with monochromatized Al Kα X–rays (energy: 1486.6 eV) at 300 W.

### 3.3. Electrochemical Characterization

N-TiO_2_ nanotubes were tested for supercapacitor and as an anode material for LIB applications. For a comparative purpose, non-doped TiO_2_ samples were subjected to above applications at the same operating conditions.

#### 3.3.1. Supercapacitor

Electrochemical supercapacitor characterizations were carried out using the AUTOLAB workstation (PGSTAT-12). Two-electrode system was employed for electrochemical measurement using swagelok-type cells. Both the working and counter electrodes were of the same active material separated by Whatman filter paper in an electrolytic solution of 1 M KOH. Cyclic voltammetric (CV) curves were obtained between the potential ranges of 0 and +0.6 V at different scanning rates (100, 200 and 500 mV s^−1^). Electrochemical impedance spectroscopy (EIS) measurement was carried out by applying a voltage of 5 mV in the frequency range between 1 Hz and 10 MHz.

#### 3.3.2. Li-Ion Battery Anode

As the active materials (TiO_2_ nanotubes and N-TiO_2_ nanotubes) were contained directly over the current collector (Ti plate), the electrodes were used as it is without binder and conducting agents. All electrodes were tested as the LIB anode in a typical CR 2032-type coin cell that was fabricated in an argon containing a MBraun glove box maintained with <1 ppm O_2_ and <1 ppm H_2_O. The electrode of dimensions (0.5 cm × 0.5 cm) containing N-TiO_2_ were used as working electrode while Li foil as counter/reference electrode, separated by Celgard, the separator soaked in 1 M LiPF_6_ (1:1 (*v/v*) EC/DEC) as electrolyte. The fabricated cells were subjected to testing at constant current density of 20 µA cm^−2^ unless otherwise mentioned in a precision battery system (Landt CT2001A, New York, NY, USA).

## 4. Conclusions

In summary, we have employed N-TiO_2_ nanotubes for enhancing the electrochemical properties of a supercapacitor and LIB. From XPS spectra, peak at binding energy of 399.8 eV ensured that nitrogen was substituted in the TiO_2_ lattice from the N 1s core level spectra. N-TiO_2_ nanotubes as a supercapacitor electrode exhibited a specific capacitance of 835 µF cm^−2^ at a scan rate of 100 mV s^−1^, which is far superior to TiO_2_ nanotubes (505 µF cm^−2^). Similarly, areal discharge capacities of 975 µA h cm^−2^ and 86 µA h cm^−2^ for N-TiO_2_ nanotubes and TiO_2_ nanotubes, respectively, were obtained as anode material for LIB. Cyclic stability and rate capability studies of N-TiO_2_ nanotubes exhibits enhanced performance compared to TiO_2_ nanotubes. As a result, the N-TiO_2_ nanotubes sample exhibits better performance, which provides suitable active materials for supercapacitor and Li ion battery applications.

## Figures and Tables

**Table 1:** Displays the current density, and specific capacitance as a function of different scan rates.

S.No.	Scan Rate (mV/s)	TiO_2_ Nanotubes		N-TiO_2_ Nanotubes	
		Current Density (µA cm^−2^)	Specific Capacitance (µF cm^−2^)	Current Density (µA cm^−2^)	Specific Capacitance (µF cm^−2^)
1 2 3	100 200 500	50.456 84.854 150.590	504.56 424.27 301.18	83.576 139.780 348.483	835.760 698.900 696.966

**Table 2:** Comparative electrochemical performance of undoped TiO_2_ - and N-doped TiO_2_ nanotubes.

S.NO	Current Density (µAcm^−2^)	TiO_2_ Nanotubes			N Doped TiO_2_ Nanotubes		
		Time (s)	Voltage (mV)	Specific Capacitance (µF cm^−2^)	Time (s)	Voltage (mV)	Specific Capacitance (µF cm^−2^)
1	80	19.6	0.606	2587.4	36.91	0.600	4921.3
2	160	5.74	0.609	1508.0	11.92	0.611	3121.4
3	240	2.70	0.649	998.4	7.17	0.614	2802.6
4	320	2.29	0.649	1129.1	5.22	0.627	2664.0

**Table 3:** The peak position of cathodic, anodic, potential difference of TiO_2_ nanotubes and N-TiO_2_ nanotubes electrodes.

S.No.	Cycle	TiO_2_ Nanotubes			N-Doped TiO_2_ Nanotubes		
		Cathodic Peak (V)	Anodic Peak (V)	Potential Difference (V)	Cathodic Peak (V)	Anodic Peak (V)	Potential Difference (V)
1	1	1.58	2.150	0.580	1.49	2.17	0.680
2	5	1.69	2.117	0.437	1.70	2.09	0.390
